# Effect of Nutmeg (*Myristica fragrans*) and Tea Tree (*Melaleuca alternifolia*) Essential Oils on the Oxidative and Microbial Stability of Chicken Fillets During Refrigerated Storage

**DOI:** 10.3390/foods13244139

**Published:** 2024-12-20

**Authors:** Sushmita Moirangthem, Gopal Patra, Subhasish Biswas, Annada Das, Santanu Nath, Arun K. Verma, Srija Pal, Niloy Chatterjee, Samiran Bandyopadhyay, Pramod K. Nanda, Geetanjali Sharma, Arun K. Das

**Affiliations:** 1 Department of Livestock Products Technology, West Bengal University of Animal and Fishery Sciences, Kolkata 700037, India; sushmitamoirangthem@gmail.com (S.M.); lptgopal81@gmail.com (G.P.); lptsubhasish@gmail.com (S.B.); dasannada.555@gmail.com (A.D.); 2 Eastern Regional Station, ICAR-Indian Veterinary Research Institute, 37 Belgachia Road, Kolkata 700037, India; santanu.vet03@gmail.com (S.N.); samiranvet@gmail.com (S.B.); npk700@gmail.com (P.K.N.); 3 Goat Products Technology Laboratory, ICAR-CIRG, Makhdoom, Mathura 281122, India; arun.lpt2003@gmail.com; 4 Laboratory of Food Science and Technology, Food and Nutrition Division, University of Calcutta, 20B, Judges Court Road, Alipore, Kolkata 700027, India; srijapal01@gmail.com (S.P.); chatterjeenil99@gmail.com (N.C.); 5 National Food Laboratory, 3, Kyd Street, Taltala, Kolkata 700016, India; geetanjali.sharma.cfl@gmail.com

**Keywords:** nutmeg essential oil, tea tree essential oil, antioxidant activity, antimicrobial properties, lipid oxidation, chicken fillets, quality and safety

## Abstract

The current study investigated the impact of nutmeg essential oil (NEO) and tea tree essential oil (TTEO) on the preservation of raw chicken fillets during nine days of refrigerated storage study. The primary aim was to explore the antioxidant and antimicrobial properties of these essential oils (EOs) and assess their ability to extend the shelf life of poultry meat. Gas chromatography–mass spectrometry (GC-MS) was utilized to identify the chemical compositions of NEO and TTEO, revealing the presence of compounds like myristicin and terpenoids, known for their antimicrobial and antioxidant activities. Antioxidant properties were evaluated using DPPH and ABTS radical scavenging assays, where both oils exhibited potent free radical scavenging abilities, with NEO showing higher efficacy than TTEO. The EOs showed their antimicrobial potential, exhibiting significant antibacterial activities against tested Gram-positive and Gram-negative pathogens, such as *Staphylococcus aureus* and *Escherichia coli*, respectively. Raw chicken fillets treated with either NEO or TTEO at 1% were analyzed for physico-chemical, microbiological, and sensory attributes. Results demonstrated that both NEO- and TTEO-treated samples maintained better microbiological qualities, with lower total viable counts and enhanced sensory attributes, such as color and odor, compared to the control samples. Furthermore, NEO and TTEO effectively delayed spoilage, extending the shelf life of chicken fillets by up to seven days. This study concludes that both the test’s essential oils can be considered natural preservatives for enhancing the safety and quality of meat.

## 1. Introduction

Poultry meat, especially chicken, offers health benefits, due to its desired nutritional contents, such as a low lipid content and a relatively high concentration of polyunsaturated fatty acids [1]. Chicken meat is also considerably popular because of its taste, ease of availability, and affordability. However, oxidation and microbial degradation are the limiting factors in the quality and acceptability of chicken meat that deteriorate the taste, color, texture, and nutritional content, leading to significant economic losses and health risks [2,3]. With increased consumer awareness and stringent food safety regulations, the imperative for ensuring the safety and quality of chicken and its products, thereby extending its shelf life, has intensified. Although the preservation methods using chemical compounds are effective, they have limitations and potential adverse health effects, prompting the exploration of natural alternatives [4,5]. Seeking to extend the shelf life of raw chicken in a sustainable way, researchers are prioritizing the exploration of natural alternatives. In this regard, EOs, derived from plants and their derivatives, and by virtue of their inherent antioxidant and antimicrobial properties, are regarded as natural alternatives. EOs are not only capable of extending the shelf life of meat and meat products, but are also effective against a variety of human ailments, thanks to their anti-inflammatory, anticancer, and antidiabetic properties [6].

In addition, EOs are approved as Generally Recognized as Safe (GRAS) by the United States Food and Drug Administration (USFDA) in foods, and also considered safe food additives in the European Union (EU) at doses lower than 2 mg/kg body weight per day [7]. Owing to their broad-spectrum antibacterial activities and high phenolic contents exhibiting strong antioxidant capacities [8], several reports utilizing EOs in extending the safety and shelf life of meat products, viz., poultry [9], pork [10], and beef [11], are available. However, there is hardly any research evaluating the efficacy of nutmeg EO and tea tree EO, as dipping solutions or marinades, in improving the quality and prolonging the shelf life of chicken fillets in refrigerated storage conditions.

Nutmeg (*Myristica fragrans*) is a widely used aromatic spice and is native to the Molucca islands of Indonesia [12]. The active components of nutmeg EO (NEO) have the potential to scavenge radicals, reduce metal ions, and inhibit lipid oxidation, with their anti-inflammatory and antimicrobial activities [13]. Likewise, tea tree (*Melaleuca alternifolia*), a member of the family Myrtaceae, is a tall shrub endemic to Australia [14]. Tea tree EO (TTEO) is well known for its medicinal properties and has traditionally been used as an antiseptic, disinfectant, and herbal medicine [15], as well as in the treatment of acne and fungal infections, such as athlete’s foot [16]. Because of its antimicrobial, antioxidant, anti-inflammatory, analgesic, and antineoplastic properties, TTEO is now being widely used in many cosmetic and pharmaceutical products, and also in the food industry [17]. 

Considering the above facts and keeping in view the potential preservative effects, the present study was undertaken to evaluate the efficacy of nutmeg and tea tree essential oils in extending the shelf life and quality of fresh chicken fillets during refrigerated storage (4 ± 1 °C).

## 2. Materials and Methods

### 2.1. Essential Oils and Chemicals

Essential oils [nutmeg (*Myristica fragrans*) and tree tea (*Melaleuca alternifolia*)], various chemicals, and reagents were purchased from standard firms like Sigma-Aldrich, Spruce Street St. Louis, MO, USA and SRL, Mumbai, India. Media for antimicrobial studies were purchased from Hi-Media, Mumbai, India and SRL, Mumbai, India.

### 2.2. Bacterial Strains

The antimicrobial activities of nutmeg essential oil (NEO) and tea tree essential oil (TTEO) were tested against four bacterial strains including *Staphylococcus aureus* (ATCC-25923), *Listeria monocytogenes* (ATCC-19111), *Escherichia coli* (ATCC-25922), and *Salmonella* Typhimurium (ATCC-14028). These bacteria were collected from the Department of Veterinary Public Health and Epidemiology at Banaras Hindu University, Mirzapur, Uttar Pradesh, India. The foodborne pathogenic bacterial cultures were maintained in brain heart infusion (BHI) broth and the cultures were incubated at 37 °C for 18–24 h under aerobic conditions, prior to use in the study.

### 2.3. Preparation of Dipping Solutions

The EO dipping solutions were prepared according to the procedure outlined by Xue and Zhong [18]. Briefly, 1 mL/100 mL solution (1%) of pure NEO and TTEO was prepared separately in two 1 L beakers by adding 5 mL of EO in 500 mL distilled water along with 50 mL of glycerol (10% *v*/*v*) as plasticizer, then mixing with 10 mL of Tween 80 (2% *v*/*v*). Solutions were homogenized with a rotor–stator homogenizer (Ika model T18, Ultra Turrax, Staufen, Germany) for 2 min. The beaker contents, along with a magnetic stir bar, were placed on a magnetic plate and stirred for 2 h for complete solubilization.

### 2.4. Antioxidant Properties of Essential Oils

#### 2.4.1. Gas Chromatography–Mass Spectrometry (GC-MS) Analysis

The phytochemical compounds present in the EOs were screened out using gas chromatography–mass spectrometry (GC-MS) triple quadrupole (GC-MS TQ8030, Shimadzu Corp., Kyoto, Japan). The Q3 scan acquisition mode was used to operate the gas chromatography attached to the Restek column (Rxi—5 ms; thickness—0.25; micron length—30 m), with start and finish times of 3 and 71 min, a scan speed of 2500, and start and end *m*/*z* values of 40 and 700, respectively. A voltage of 70 eV was used for ionization. The components were identified by comparing retention times and fragmentation patterns with mass spectra found in the National Institute of Standards and Technology (NIST) spectral library, which was saved in the GC-MS/MS computer program [19].

#### 2.4.2. Total Phenolics Content (TPC)

The Folin–Ciocalteu (F-C) technique was used to determine the TPC of EOs, as described by Sánchez-Rangel et al. [20]. To 100 μL of various EO dilutions, approximately 0.75 μL of F-C reagent was added, and the final volume was made up to 10 mL with distilled water (DW). After 5 min, 750 μL of sodium carbonate solution (7.5%) was added and the tubes were kept for 90 min in the dark at an ambient room temperature, and the absorbance was measured at 725 nm in comparison to a blank. The TPC was determined as gallic acid equivalents (GAE), in mg/g of the oil, by drawing a standard curve using various gallic acid concentrations.

#### 2.4.3. Total Flavonoid Content

Total flavonoids were determined by the aluminum chloride (AlCl_3_) colorimetric method [21]. The EOs were diluted serially to make various concentrations. From each concentration, 1 mL sample was added to the test tube containing 4 mL of distilled water. Meanwhile, 0.3 mL of 5% NaNO_2_ was added to the test tube, to which 0.3 mL of 10% AlCl_3_ was added. After 6 min, 2 mL of 1 M NaOH was added to the mixture. 4.4 mL of distilled water was added immediately thereafter to make the final volume 10 mL, and the absorbance reading was taken at 510 nm using a spectrophotometer (Eppendorf BioSpectrometer, Westbury, NY, USA). The total flavonoid concentration was determined by calculating the average absorbance value from three separate readings. The linear equation derived from the standard calibration curve was utilized to express the flavonoid concentration in terms of catechin equivalent (mg CE/g).

#### 2.4.4. Diphenyl 2-Picrylhydrazyl (DPPH) Radical Scavenging Activity

The DPPH radical scavenging activity (RSA) of the EOs was determined as per the standard procedure [22], with slight modification. Different concentrations (0.2, 0.4, 0.6, 0.8, and 1.0 mL) of EOs (1 mg/mL) were added to test tubes and each volume was made up to 4 mL with distilled water. Then 1 mL of 1 mM DPPH methanolic solution was added to each test tube. All the test tubes were shaken well and kept for 30 min. Control was prepared by adding 1 mL of DPPH solution and 4 mL of DW, and absorbance was taken immediately at 517 nm (DW used as blank) using a spectrophotometer (Eppendorf BioSpectrometer, USA). The RSA was measured using the following formula:

RSA (%) = (Absorbance Control − Absorbance Sample/Absorbance Control) × 100

The IC_50_ values were calculated from the scavenging percentage (RSA%) of radical formation in the presence of the test substances.

#### 2.4.5. Azinobis (3-Ethylbenzothiazoline-6-Sulonic Acid) (ABTS) Radical Scavenging Activity

The ABTS·+ scavenging activities of EOs were measured by the standard method [23]. ABTS was dissolved in water to a 7 mM concentration. ABTS·+ was produced by reacting ABTS stock solution with 2.45 mM potassium persulphate (final concentration) and allowing the mixture to stand in the dark at room temperature for 16 h before use. The radical was stable in this form for more than 2 days, when stored in the dark at an ambient temperature. The ABTS·+ solution was then diluted with methanol to an absorbance of 0.850 ± 0.05 at 734 nm. A volume of 3.0 mL of this ABTS·+ solution was added to 1.0 mL of different concentrations of the methanolic extract and incubated for 4 min at an ambient temperature. After that, absorbance was observed at 734 nm. The percentage of inhibition was calculated using the following formula: inhibition (%) = (A_0_ − A_1_/A_0_) × 100, where A_0_ is the absorbance of control and A_1_ is the absorbance of sample. The IC_50_ value was also determined using a standard curve.

### 2.5. Antimicrobial Activities of Essential Oils

#### 2.5.1. Agar Well Diffusion Assay

The antibacterial activity of EOs was tested using the agar diffusion method, with slight modification of the method, as described by Singh et al. [24]. Briefly, all the microbial cultures were adjusted to 0.5 McFarland standards, which is visually comparable to a microbial suspension of approximately 1.5 × 10^8^ CFU/mL. Mueller−Hinton agar in the petri plates was allowed to solidify. Bacterial cultures were sampled with the help of a sterile cotton swab. A sterile crock borer was used to make a 6 mm well in the agar. The base of each well was sealed with 50 μL of sterilized molten nutrient agar. The wells were then loaded with 100 μL volume of different concentrations of essential oils and the petri plates were incubated at 37 °C for 24 h. The antimicrobial activities of the EOs were evaluated by measuring the zone of inhibition (ZOI) against the test microorganisms. The ZOI was measured in mm, including the well diameter (6 mm).

#### 2.5.2. Estimation of Minimum Inhibitory Concentration (MIC)

To evaluate the MIC of EOs, the resazurin-based microdilution method was followed [25]. Briefly, the inocula of each test bacteria were prepared in phosphate buffered saline (PBS), following the Clinical and Laboratory Standards Institute’s (CLSI) guidelines (http://clsi.org/, accessed on 1 December 2023), where the OD600 value (0.08–1.12) was adjusted, resulting in ~1 × 10^8^ CFU/mL. Then, the adjusted inocula were further diluted in PBS, resulting in ~1 × 10^6^ CFU/mL. Different concentrations of the EOs were prepared by dilution in PBS from the initial parent stock. Each well in column 1 was dispensed with 100 μL Luria broth (LB) for *S.* Typhimurium, *E. coli*, *S. aureus*, and Brain heart infusion (BHI) broth for *L. monocytogenes*. About 2.5 μL of respective culture was added to each well. Then, each well in column 2 to 9 contained 100 μL of LB or BHI. Next, 100 μL of different concentrations of EOs prepared was added to columns 2 to 9 and 2.5 μL of respective culture was added in each well. Column 11 had 100 μL of LB/BHI and 100 μL of extract, which served as negative control (NC). The inoculated plates were incubated at 35 °C for 24 h. Following incubation, 20 μL of sterile resazurin dye (0.015% *w*/*v*) was dispensed into each well of columns 1 to 9 and 11, and then plates were incubated for 1–2 h to observe color change. Following incubation, the blue resazurin color that remained unchanged in the columns with the lowest concentration was reported as the MIC.

### 2.6. Chicken Fillet Preparation and Analysis of Meat Samples

For this study, birds were purchased from the local market and slaughtered and dressed in the poultry processing unit of the Department of Livestock Products Technology, WBUAFS, Kolkata, West Bengal, as per the standard method [26]. After scientific and hygienic dressing, skinless whole chicken breast muscle was separated and deboned. Chicken fillets were separated into three groups and dipped in solution for 30 min and marked as follows: C—fillets immersed in distilled water; T_1_—fillets immersed in 1% NEO; T_2_—chicken fillets immersed in 1% TTEO. After draining out the dipping solution completely, the chicken fillets were packed aerobically in low density polyethylene (LDPE) bags, sealed and stored at 4 ± 1 °C for analysis of the different quality parameters on the 1st, 3rd, 5th, 7th, and 9th day of storage.

#### 2.6.1. Proximate Composition

Proximate composition, such as crude protein, moisture, ether extract, and ash value were determined by standard methods [27] on the day of processing (0 day).

#### 2.6.2. pH

A combination electrode digital pH meter (Benchtop pH meter, Br Biochem, PHS-25CW, New Delhi, India) was used to determine the pH of each individual meat sample. Briefly, 50 mL of distilled water was used to homogenize 10 g of sample for approximately a minute, using a tissue homogenizer (Omni, Kennesaw, GA, USA). After allowing the homogenized material to stand for 5 min, it was shaken once again using a glass rod. By directly submerging the electrode in the suspension, the pH was measured.

#### 2.6.3. Water Holding Capacity (WHC)

Water holding capacity (WHC) of chicken fillet was measured by the filter paper press method [28], with slight modification. About 500 mg of meat sample was pressed between folded filter papers by applying 2.8 kg weight for 5 min. The weight of meat flake and filter paper after pressing was recorded. The WHC was expressed as the percentage of water retained by the meat sample. WHC (%) = 100 − (B − F/B × 100), where B = weight of meat sample and F = weight of meat sample after pressing + weight of difference in the weight of filter paper.

#### 2.6.4. Thiobarbituric Acid Reacting Substance (TBARS) Value

Lipid oxidation was estimated by measuring the TBARS value, determined as per the standard procedure [29] with slight modifications. With the use of a tissue homogenizer (Omni, Germany), 2 g of meat sample was homogenized in 10 mL of cooled 20% TCA for 2 min. After that, the sample was left to stand for 10 min. The homogenate was filtered through a Whatman Filter Paper No.1, from which 3 mL of filtrate was taken out in a separate test tube, and to this 3 mL of 5 mM TBA reagent was added and mixed well. The tubes were placed for 35 min in a boiling water bath. The samples were allowed to cool, and a reading was taken for optical density at 530 nm. The absorbance value was multiplied by a factor of 5.2 to determine the TBA value, which was expressed as mg malonaldehyde per kg (mg MDA/kg) of samples.

#### 2.6.5. Instrumental Color Values

Color profile analysis was conducted using the Lovibond Tintometer (Lovibond RT-300, Reflectance Tintometer, United Kingdom) set at 2° of cool white light (D65) and known as L*, a*, and b* values. Samples were ground in the mixer, placed in the sample holder, and secured against the viewing aperture. The L* value (brightness = 100 or lightness = 0), a* (+ redness/− greenness), and b* (+ yellowness/− blueness) values were recorded from the surface of petri plates uniformly filled with ground meat samples [30].

#### 2.6.6. Microbiological Quality

Meat samples were analyzed for microbial quality on the 1st, 3rd, 5th, 7th, and 9th day of refrigerated storage [31], and the following parameters were monitored: total viable counts (TVCs), *S. aureus*, *E. coli*, *Salmonella* spp., and total coliform counts. Chicken sample (10 g) from each treatment group was aseptically transferred into separate stomacher bags (Seward Medical, UK), containing 225 mL of sterile buffered peptone water (BPW) solution (0.1 g/100 mL), and homogenized in the stomacher (Lab Blender 400, Seward Medical, Worthing, West Sussex, UK) for 60 s. For each sample, serial decimal dilutions were prepared in BPW solution (0.1 g/100 mL). 1 mL of these serial dilutions of chicken homogenates was poured onto the sterile petri plates and mixed with the respective agar. The TVC was determined using plate count agar after incubating for 48 h at 37 °C. The *S. aureus* count was determined using Baird–Parker agar, whereas the *Salmonella* spp. count was determined using Hektoen Enteric agar after incubation at 37 °C for 24 h. The *E. coli* and total coliform count were determined using Chromocult coliform agar after incubation for 48 h at 37 °C. All the plates were examined visually for the colony types and morphological characteristics associated with each growth medium. Microbial colonies were counted and expressed as log_10_ CFU/g chicken meat.

#### 2.6.7. Sensory Evaluation

A sensory evaluation of the control samples and experimental chicken breast fillets, for two sensory attributes like color and odor, was performed by semi-trained panelists. The fillets were evaluated for color and odor using a 5-point descriptive scale [32], where 1—very undesirable/unattractive/dull, 2—undesirable, 3—moderately desirable, 4—desirable, 5—very desirable.

### 2.7. Statistical Analysis

The study was conducted three times, with each replication involving the measurement of every parameter in duplicate (n = 6), except for sensory evaluation (n = 10). The analysis was conducted with SPSS software (Version 20.0), and a two-way ANOVA was used to analyze the data from the storage study. The means were compared using Duncan’s multiple range test, and the significance level was assessed at a 95% confidence interval. The values were presented as mean along with standard error (Mean ± SE).

## 3. Results and Discussion

### 3.1. Antioxidant Properties of Essential Oils

#### 3.1.1. Chemical Composition of NEO and TTEO Using GC-MS

**Figure 1:**
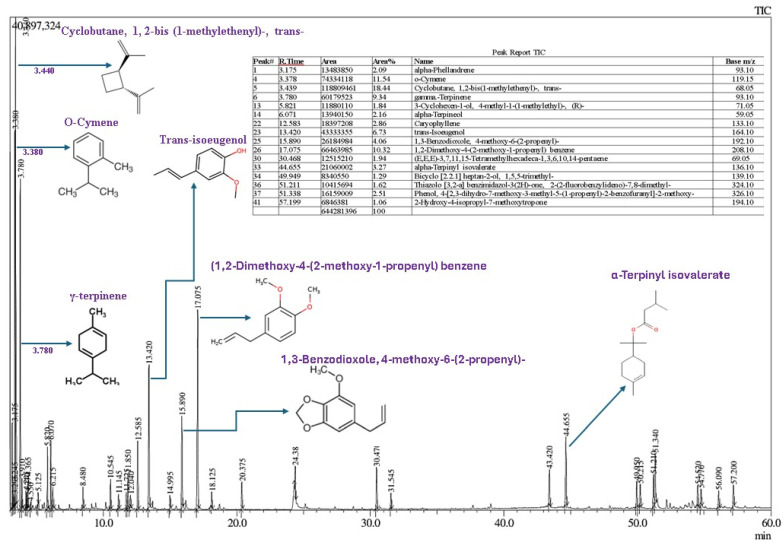
Gas chromatography and mass spectrometry analysis of nutmeg essential oil with major compounds.

The GC-MS chromatogram of NEO indicated 41 compounds (Figure 1). Key components included cyclobutane, 1,2-bis(1-methylethenyl)-, trans- (18.44%), o-cymene (11.54%), and 1,2-dimethoxy-4-(2-methoxy-1-propenyl) benzene (10.32%). Terpenes and their derivatives were prominent, with notable compounds like γ-terpinene (9.34%), 1,3-benzodioxole, 4-methoxy-6-(2-propenyl)- (4.06%), alpha-terpinyl isovalerate (3.27%), caryophyllene (2.86%), alpha-terpineol (2.16%), alpha-phellandrene (2.09%), myrtenol (0.83%), safrole (0.74%), 3-carene (0.62%), and 4-carene (0.28%). Although most of the components found are in agreement with previous studies [33,34], the variations in concentrations of key EO components could be due to differences in agro-climatic conditions, plant varieties, and analytical methods [12].

**Figure 2:**
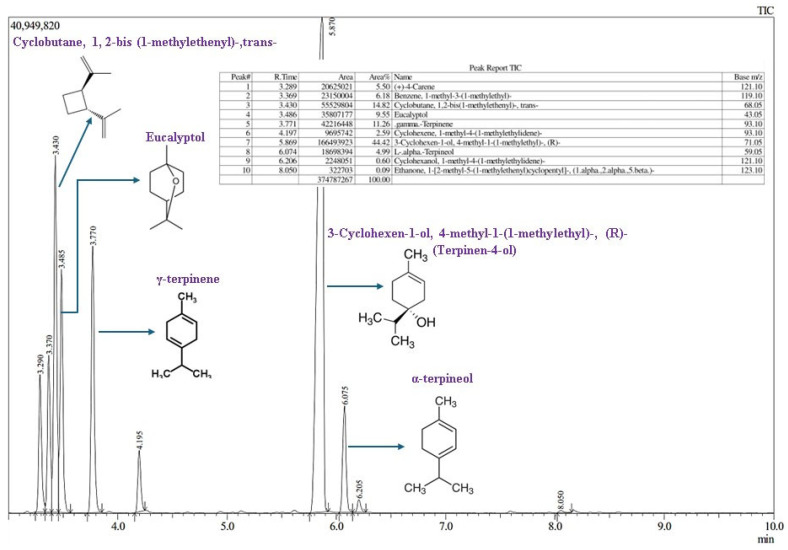
Gas chromatography and mass spectrometry analysis of tree tea essential oil with major compounds.

The GC-MS analysis of TTEO identified and quantified 10 compounds (Figure 2). The dominant component was 3-cyclohexen-1-ol, 4-methyl-1-(1-methylethyl)-, and (R)-(terpinene-4-ol) (44.42%), followed by trans-cyclobutane, 1,2-bis(1-methylethenyl)- (14.82%), γ-terpinene (11.26%), eucalyptol (9.55%), and others. The principal constituent 3-cyclohexen-1-ol, 4-methyl-1-(1-methylethyl)-, and (R)-, a key terpene and alcohol, contributes to the oil’s antioxidant properties. This finding is consistent with a previous study that identified terpinen-4-ol as the major component of the total oil content, at 41.11% [35].

#### 3.1.2. Total Phenolics Content

In this study, the TPC of NEO was 64.51 ± 0.82 mg GAE/g, while TTEO exhibited a higher TPC value of 78.21 ± 0.75 mg GAE/g (Table 1). Several studies have quantified the TPC in NEO and TTEO, yielding varying results. Soysa et al. [36] reported TPCs of 28.4 ± 0.3 mg GAE/g dry weight in nutmeg mace, and 34.8 ± 0.4 mg GAE/g dry weight in nutmeg seed, which were lower than the values found in the present study. Gupta et al. [37] evaluated the antioxidant activity of nutmeg seed extracted with various solvents, and found that nutmeg seed ethanol extract exhibited a TPC of 70.69 ± 2.06 mg GAE/100 g. As far as TTEO is concerned, Badr et al. [35] reported TPCs of 0.62 mg of GAE/g of oil, while TTEO nanoemulsion had a TPC of 0.64 mg of gallic acid equivalent/g of oil, which were lower than the values obtained in the present study. Another species used for TTEO production, *Melaleuca bracteata*, exhibited a higher concentration of phenolics, i.e., 110 mg/g of dry weight [38].

#### 3.1.3. Total Flavonoid Content

Flavonoids represent one of the most diverse and widespread groups of natural compounds, encompassing flavones, isoflavones, flavonols, anthocyanins, and catechins, which are recognized as significant natural phenolics [39]. In this study, the total flavonoid contents (TFC) of NEO and TTEO were measured at 9.38 ± 0.27 mg quercetin equivalent (QE)/g and 17.29 ± 0.31 mg QE/g, respectively (Table 1). Recent studies have also shown that the flavonoid content of NEO derived from seed powder (180 μg quercetin equivalent/100 g of dry weight) exceeded that of the crude extract of nutmeg (21 μg quercetin equivalent/100 g of dry weight) [34]. Additionally, Antasionasti et al. [40] reported a TFC of 26.929% w/w quercetin equivalent in NEO. A higher level of flavonoids, 951 ± 4.50 mg/mL, was also found in *Melaleuca bracteata* [38]. Conversely, Badr et al. [35] revealed TFC values of 0.10 mg rutin equivalents/g in tea tree oil and 0.08 mg rutin equivalents/g in tea tree oil nanoemulsion.

#### 3.1.4. DPPH Radical Scavenging Activity

The DPPH compound serves as a widely utilized free radical compound for assessing the free radical scavenging capacity of various extracts. A lower IC_50_ value is indicative of a higher antioxidant activity of essential oils [41]. In the DPPH radical scavenging activity assay, NEO and TTEO demonstrated robust antioxidant activity, with IC_50_ values of 2.94 ± 0.18 µg/mL and 12.68 ± 0.24 µg/mL, respectively (Table 1). The results revealed that NEO exhibited a higher scavenging activity compared to TTEO. These findings align with the literature data of Adiani et al. [42], who reported a Trolox equivalent antioxidant capacity (TEAC) for NEO of 2.94 ± 0.09 μM g^−1^. Similarly, Sohilait et al. [43] documented a comparable DPPH radical scavenging activity of nutmeg seed essential oil, with an IC_50_ value of 2.48 ± 0.42 µg/mL, compared to nutmeg mace oil, which exhibited an IC_50_ value of 2.80 ± 0.24 µg/mL. Significantly stronger antioxidant activity in NEO with an EC_50_ value of 11.07 ± 0.06 mg/mL was also reported [44]. Additionally, the GC-MS analysis of NEO revealed the presence of safrole and myristicin, which are crucial for the oil’s antioxidant properties [45]. Vyshak et al. [46] found that the nutmeg fruit flesh’s essential oil exhibited a DPPH radical scavenging activity of 66.66%.

These results are also consistent with the previous work of Noumi et al. [41], who reported an IC_50_ value of 12.5 µg/mL, exhibiting the promising antioxidant abilities of *M. alternifolia* essential oil. Similarly, the antioxidant concentration of *M. alternifolia* tea tree oil, at which 50% of the reaction was inhibited (IC_50_), was reported to be 12.5 µg/mL [47]. Yasin et al. [38] reported that the average DPPH scavenging activity of *M. bracteata* tea tree oil was 86.848 ± 2.43 mg/mL.

#### 3.1.5. ABTS Radical Scavenging Activity

In ABTS+ assay, the antioxidant activity was evaluated by measuring the reduction in ABTS+ radicals by antioxidants, with results expressed as IC_50_, representing the concentration of the extract required to scavenge 50% of the ABTS radicals. In this case, NEO exhibited an IC_50_ value of 1.26 ± 0.22, while TTEO showed an IC_50_ value of 8.62 ± 0.55 for ABTS radical cation scavenging activity (Table 1). Notably, NEO displayed higher ABTS cation scavenging activity compared to TTEO. Consistent with these findings, Sohilait et al. [43] similarly reported the ABTS scavenging activity of both nutmeg seed EO and nutmeg oil, with IC_50_ values of 1.09 ± 0.25 µg/mL. In contrast, Antasionasti et al. [40] reported a less potent antioxidant capacity of *M. fragrans* water extract, with an IC_50_ value of 89.98 ± 0.480 μg/mL. Badr et al. [35] also reported an antioxidant activity of 28.8 ± 0.8 TEAC (μmol/g) for nutmeg through ABTS assay. Such variability in scavenging activity may be influenced by factors such as the geographical origin of the sample, the solvent used, and the extraction method.

With regard to TTEO, numerous studies have reported its antioxidant activity, with varying results. In our study, the ABTS scavenging activity of the oil was lower than in the findings of Yang et al. [48], whose data demonstrated that TTEO showed an RC_50_ value of 1.6 ± 0.02% against ABTS radicals. Similarly, Zhao et al. [49] demonstrated the IC_50_ for ABTS^+^ scavenging activity of tea tree oil to be 3.5 × 10^−2^ mL/mL.

### 3.2. Antimicrobial Activities of Essential Oils

#### 3.2.1. Agar Well Diffusion Assay

**Figure 3:**
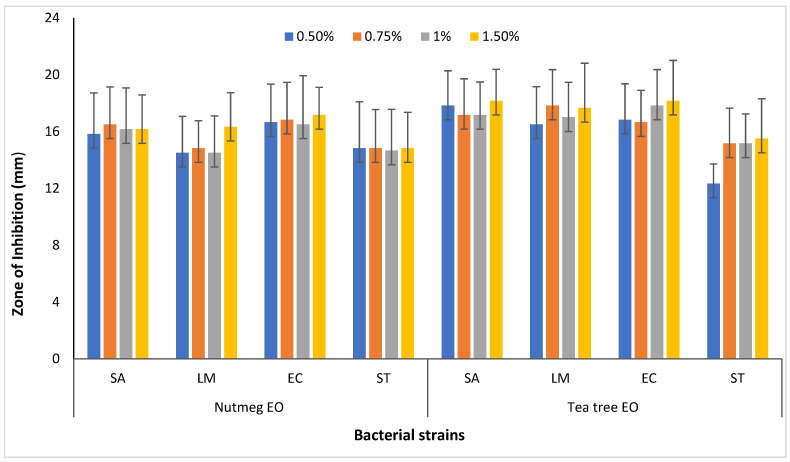
Zone of inhibition of NEO and TTEO by agar well diffusion method against different pathogenic bacterial strains (SA—*Staphylococcus aureus*; LM—*Listeria monocytogenes*; EC—*Escherichia coli*; ST—*Salmonella* Typhimurium).

The observed zone of inhibition (ZOI) in mm at different concentrations of NEO and TTEO is presented in Figure 3. The EOs (NEO and TTEO) at different concentrations (0.5%, 0.75%, 1%, and 1.5%) showed good antibacterial activity against all four bacterial strains. Gram-positive bacteria were more sensitive than Gram-negative bacteria for TTEO, whereas the reverse was found for NEO.

#### 3.2.2. Determination of Minimum Inhibitory Concentration (MIC)

In this study, the MIC of NEO was found to be 15 μL/mL against *Staphylococcus aureus*, *Escherichia coli*, *Listeria monocytogenes*, and *Salmonella* Typhimurium. On the other hand, TTEO displayed MIC values of 100 μL/mL against *L. monocytogenes*, whereas it was 15 μL/mL against *S. aureus*, 25 μL/mL against *E. coli*, and 50 μL/mL against *S.* Typhimurium. NEO demonstrated an MIC of 10 μL/mL against *E. coli* O157:H7 [50]. Our research findings are in agreement with those of Cui et al. [51], who reported that nutmeg oil exhibited equal inhibitory activity against both Gram-positive and Gram-negative bacteria, with an MIC of 0.05%. However, Bharti et al. [19] observed a relatively higher inhibition of Gram-positive bacteria by essential oils compared to Gram-negative bacteria, contrasting with the MIC values recorded for NEO in this study. This difference could be attributed to bacterial tolerance and the composition of phytochemical compounds present in the essential oil.

The antimicrobial effectiveness of TTEO may be attributed to the hydrophobic nature of its terpenes. Main components such as terpinen-4-ol and 1.8-cineole are responsible for damaging the cytoplasmic membrane of Gram-positive bacteria, resulting in both bacteriostatic and bactericidal effects. Conversely, the lipopolysaccharide layer in the outer membrane of Gram-negative bacteria limits the diffusion of hydrophobic components [52]. The MIC of TTEO against *L. monocytogenes* differed from that reported by Mazzarrino et al. [53], who found an MIC of 10 μL/mL. This variance could be attributed to differences in the concentration of terpinen-4-ol (43%) in the oil used.

Basically, when antimicrobial compounds are present in the environment, microorganisms may respond by adjusting the synthesis of fatty acids and membrane proteins to modify the fluidity of their cell membranes. Essential oils (EOs) and their components, due to their hydrophobic nature, are able to penetrate the lipid bilayer of bacterial membranes [52]. Once inside, EOs can affect both the permeability and functionality of membrane proteins. Some essential oils, particularly those rich in phenolic compounds, can integrate into the phospholipid bilayer of bacterial cell walls, binding to proteins and disrupting their normal functions. This suggests that the bacterial membrane is the primary target for the actions of EOs.

### 3.3. Proximate Composition of Chicken Fillets

The moisture, crude protein, crude fat, and total ash percentages of treated raw chicken fillets (T1-NEO and T2-TTEO) and control (C) on the day of processing (day 0) are shown in Table 2.

#### 3.3.1. Moisture

The mean moisture contents (%) of chicken fillets were 73.77 ± 0.73, 73.98 ± 0.71, and 73.92 ± 0.51 for control, T1, and T2, respectively (Table 2). The essential oils exhibited no significant effect (*p* > 0.05) on the moisture content of the chicken fillets. These findings are in agreement with those observed by Yaghoubi et al. [54], where it was demonstrated that chitosan coating (1%) containing *Artemisia fragrans* essential oil had no significant effect on the moisture content of fresh chicken meat. Similarly, Kiarsi et al. [55] reported a non-significant (*p* > 0.05) difference in the moisture content of beef slices treated with NEO-loaded sage seed mucilage edible coating, compared to the control samples.

#### 3.3.2. Crude Protein

The crude protein contents (%) of chicken fillets were 21.55 ± 0.43, 21.61 ± 0.67, and 21.63 ± 0.36 for control, T1, and T2, respectively (Table 2). No significant difference (*p* > 0.05) was observed in the protein content of treated chicken fillets (T1 and T2) compared with control. Yaghoubi et al. [54] also reported similar results on the crude protein content of fresh chicken fillets, where no significant change was observed after treatment with chitosan coating (1%) containing *A. fragrans* essential oil.

#### 3.3.3. Crude Fat

The crude fat contents of chicken fillets were 2.52 ± 0.14, 2.49 ± 0.04, and 2.54 ± 0.02 for control, T1, and T2, respectively (Table 2). No significant difference (*p* > 0.05) in the crude fat content was found in the treated fillets (T1 and T2) in comparison with control. In line with the results of this study, Yusri et al. [56] observed no significant difference (*p* > 0.05) in the fat content of goat meat supplemented with 5% of nutmeg leaves, in comparison with control. Similar results were also observed in fresh chicken meat, where chitosan coating (1%) containing *A. fragrans* essential oil had minimal effect on the crude fat content [54].

#### 3.3.4. Total Ash

The total ash contents of chicken fillets were 1.45 ± 0.28, 1.41 ± 0.26, and 1.49 ± 0.35 for control, T1, and T2, respectively (Table 2). A non-significant (*p* > 0.05) change in the ash content of chicken fillets was observed in the treatment groups compared with control. In a study, Dzudie et al. [57] reported that there was no effect (*p* > 0.05) on the ash content of raw and cooked beef patties with the incorporation of different oils, viz., groundnut oil, maize oil, 0.2% (*w*/*w*) each of ginger essential oil and 0.2% (*w*/*w*) basilica essential oil. Yaghoubi et al. [54] also found no significant difference in the ash content of raw chicken meat with chitosan coating (1%) containing *A. fragrans* essential oil.

### 3.4. Physico-Chemical Qualities of Chicken Fillets

#### 3.4.1. pH

The pH values of chicken fillets of both the treated (T1-NEO and T2-TEO) and the control group during 9 days of refrigeration storage study are presented in Table 3. On day 1, the pH values of raw chicken fillets were 5.72 ± 0.01 for control, 5.73 ± 0.02 for T1, and 5.71 ± 0.02 for T2. By day 9, the pH values for control, T1, and T2 were 7.42 ± 0.04, 6.87 ± 0.01, and 6.86 ± 0.01, respectively. A gradual increase in pH was observed for all the groups over the storage period. This increase can be attributed to protein decomposition under the action of microorganisms, leading to the production of small alkaline nitrogen-containing molecules, such as ammonia and trimethylamine, thereby causing an elevation in pH [58]. However, the pH values of treatment groups (T1 and T2) and their rate of increase were significantly (*p* < 0.05) lower than those of the control samples, indicating that the EOs had a certain effect in slowing down the protein degradation process and extending the shelf life of chicken fillets.

Our results are consistent with the findings of Wei et al. [58], who observed the preservative effect of compound spice essential oils, including TTEO, on marinated chicken. Similarly, Kiarsi et al. [55] noted similarities in the pH trend of coated beef slices treated with NEO and sage seed mucilage during 6 days of refrigerated storage. Additionally, Silva et al. [59] reported that *M. alternifolia* EO was effective in containing spoilage in ground beef throughout storage, as the pH did not significantly differ over the studied period.

#### 3.4.2. Water Holding Capacity

The results of the WHC of samples treated with NEO and TTEO dipping marinades are presented in Table 3 and compared with control. On day 1, the WHC values of raw chicken fillets were 73.84 ± 2.06%, 73.75 ± 1.12%, and 73.80 ± 0.48% for the control, T1, and T2 respectively. By day 9, these values decreased to 61.35 ± 0.55%, 67.04 ± 1.40%, and 68.21 ± 1.60% for the control, T1, and T2, respectively. Although no significant difference (*p* > 0.05) was observed between treated (T1 and T2) and control, a slightly higher WHC was noted in the treated samples from day 3 until the end of the storage period. Essential oils are reported to be effective in protecting proteins in meat from oxidation and microbial decomposition, and also enhancing protein–protein interactions, thereby increasing water holding capacity [60]. The decrease in WHC values during storage indicates changes in the free water content, resulting from alterations in myofibrillar proteins and structure. This reduction in free water could also be attributed to purge loss experienced during storage [61]. Similar findings were reported by Zhu et al. [62], who observed an improved WHC in pork tenderloin meat batters treated with NEO encapsulated in solid liposomes. Conversely, Deminicis et al. [63] found no significant difference in the WHC of meat from quails supplemented with dietary *Mentha piperita* and *M. alternifolia* essential oils.

#### 3.4.3. TBARS Value

**Figure 4:**
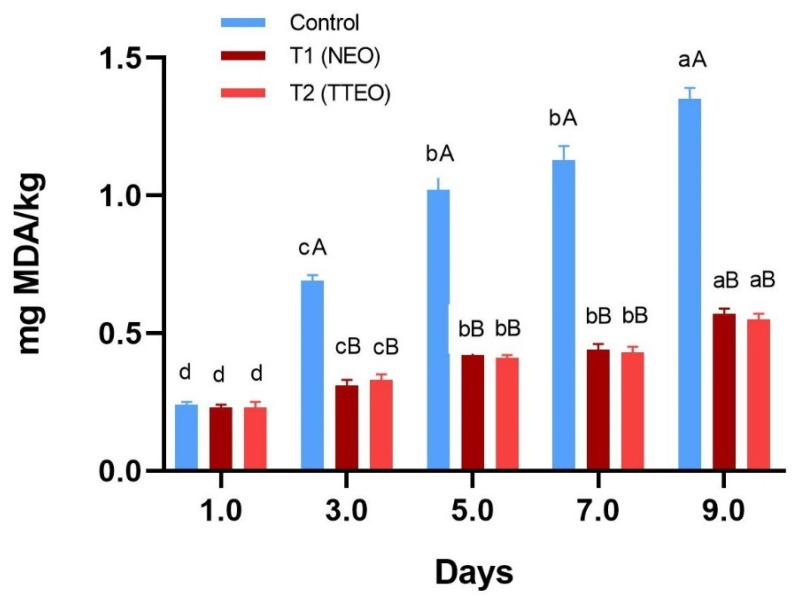
Effect of essential oils on TBARS values of chicken fillets during storage at 4 °C. Bars with different superscript (a–d) between days and (a–c) between treatments differ significantly (*p* < 0.05).

The findings of thiobarbituric acid reactive substances (TBARS) in treated (T1-NEO and T2-TTEO) and control samples are presented in Figure 4. On day 1, the mean TBARS values of raw chicken fillets were 0.24 ± 0.01 mg malonaldehyde per kg, 0.23 ± 0.01 mg/kg, and 0.23 ± 0.02 mg/kg for the control, T1, and T2, respectively. By day 9, these values increased to 2.35 ± 0.04 mg/kg, 1.57 ± 0.02 mg/kg, and 1.55 ± 0.02 mg/kg for the control, T1, and T2, respectively. Although all samples exhibited an increasing trend in TBARS values throughout the storage period, the control samples showed significantly (*p* < 0.05) higher TBARS values from day 3 of refrigerated storage onwards, indicating spoilage by day 5 compared to the treated samples.

In comparison, NEO-treated fillets exhibited lower TBARS values, indicating maintained oxidative stability by inhibiting the lipid oxidation. In fact, nutmeg contains antioxidant properties, including isoeugenol, eugenol, and β-carophyllene, which interact strongly with lipid bilayers, thereby enhancing antioxidant content. Compounds such as benzyl and aromatic compounds found in NEO possess effective hydrogen abstraction abilities from free radicals [37]. Our findings also align with a study conducted by Zakaria et al. [64] on raw beef treated with nutmeg extract during frozen storage. Additionally, results from this study are consistent with those of Cai et al. [65], where pork and chicken samples treated with gliadin/gum arabic–tea tree essential oil nanofibers exhibited lower TBARS values compared to the untreated group during storage (*p* < 0.05).

Likewise, the reduced TBARS values in TTEO-treated samples may be attributed to compounds like α-terpineol, terpinolene, and γ-terpineol present in tea tree oil, which provide hydrogen atoms for electrons and stabilize free radicals [66]. Tea tree oil has also been suggested as an alternative for maintaining oxidative stability in food matrices and exhibits strong free radical scavenging abilities, inhibiting lipid peroxidation [47].

### 3.5. Instrumental Color Values

The mean lightness (L*), redness (a*), and yellowness (b*) values of chicken fillet samples treated with NEO and TTEO during 9 days of refrigeration storage are illustrated in Table 4. The mean lightness (L*) values of control, T1, and T2 ranged from 56.98 ± 2.06 to 42.70 ± 0.44, 56.78 ± 1.95 to 52.53 ± 1.70, and 55.94 ± 2.02 to 52.30 ± 1.43, respectively, from day 1 to day 9 of refrigerated storage (Table 4). Initially, lightness (L*) values were not significantly affected (*p* > 0.05) during the first three days of storage. However, during the subsequent storage period (5th, 7th, and 9th days), L* values decreased significantly (*p* < 0.05) for the control group, while there was a non-significant decrease in L* values for the essential oil-treated (T1 and T2) samples. This may be attributed to alterations in light absorption or scattering properties caused by pigments present in the essential oil [67]. Behbahani et al. [68] reported a similar decrease in L* values during storage in minced meat treated with 0.5% to 2% cumin essential oil-loaded edible coating. Similarly, a decrease in L* values in chicken breast meat was documented by Jaspal et al. [69] upon treatment with 0.2% oregano essential oil and 1.25% lactic acid.

The mean redness (a*) values for control, T1, and T2 ranged from 8.65 ± 0.53 to 2.91 ± 0.25, 8.59 ± 0.34 to 6.38 ± 0.28, and 8.42 ± 0.32 to 7.09 ± 0.21, respectively, from day 1 to day 9 of refrigerated storage (Table 4). During the first three days of storage, the redness values (a*) were not significantly affected (*p* < 0.05) in all the samples. Although the a* values decreased significantly (*p* < 0.05) during the subsequent storage period (5th, 7th, and 9th days), they did so at a lower rate for the treated samples (T1 and T2) compared to the control. Such smaller changes in the a* values for treated samples could be due to the presence of antioxidant compounds in EOs, such as phenols, which protect against oxidative processes that can cause meat discoloration [70]. In fact, high a* values are associated with the presence of oxymyoglobin, while a reduction in a* values is related to the formation of metmyoglobin, which darkens the muscle of meat [71].

Different researchers have emphasized the role of essential oils in maintaining the a* values of meat products. In a study, Parvin et al. [72] highlighted the effect of nutmeg in maintaining the redness of meat products. Additionally, minimal change in the a* values of ground goat meat treated with 1% nutmeg extract was reported by Nishad et al. [73]. Krichen et al. [74] also noted similar results in ground beef meat treated with 0.1% and 1.5% pistachio by-product EO. A similar decline in the redness (a*) values during storage at 4 °C was previously observed for minced meat wrapped in 4% *Rosmarinus officinalis* EO-supplemented active packaging [70]. These findings align with those of Befa Kinki et al. [67], who reported a significant decrease in the redness (a*) values of raw minced meat samples throughout the storage period.

The yellowness (b*) value is correlated with the formation of metmyoglobin. In this study, the mean yellowness (b*) values for control, T1, and T2 ranged from 9.67 ± 0.21 to 12.76 ± 0.61, 9.65 ± 0.30 to 10.74 ± 0.57, and 9.58 ± 0.26 to 10.72 ± 0.32, respectively, from day 1 to day 9 of refrigerated storage (Table 4). The yellowness values (b*) of treated samples (T1 and T2) were not significantly affected, although a slight increase was observed during storage. Similarly, Jaspal et al. [69] noted comparable b* values in chicken breast meat treated with 0.2% oregano EO and 1.2% lactic acid, during storage in modified atmosphere packaging and oxygen-permeable packaging at 4 °C. Papazoglou et al. [75] found constant b* values for chicken liver meat treated with 0.1% and 0.3% thyme EO throughout 12 days of storage at 4 °C in vacuum packaging.

### 3.6. Microbiological Qualities of Chicken Fillets

#### 3.6.1. Total Viable Counts (TVCs)

The TVC of the control sample increased from 4.91 ± 0.36 on day 1 to 7.94 ± 0.17 log CFU/g on day 7 of storage, and by day 9, the colonies surpassed the countable range, as the sample was severely spoiled. Meanwhile, the mean TVC values for T1 and T2 ranged from 3.27 ± 0.23 to 5.51 ± 0.18 log CFU/g and 3.17 ± 0.26 to 5.81 ± 0.37 log CFU/g, respectively, during 1–9 days of refrigerated storage (Table 5). Notably, the TVC of treatment samples (T1 and T2) was significantly lower (*p* < 0.05) compared to the control samples at all storage intervals, with no significant difference (*p* > 0.05) between both. Furthermore, it was observed that the TVC values of T1 and T2 remained slightly below the standard threshold limit of 6–7 log CFU/g until the 9th day of storage [76], whereas the control samples exceeded acceptable limits even on the 5th day, indicating early spoilage. From the above results, it can be deduced that the essential oils not only exhibited inhibitory effects against microbial growth, but also contributed to maintaining the quality and stability of the meat samples.

The reduced TVC and extended shelf life, as observed in treated chicken fillet samples, are likely due to the antimicrobial effects or remarkable antibacterial activity of essential oils, as reported in various studies [35,77]. Consistent with this study, Wei et al. [58] reported a lower total number of colonies (TNC) in marinated chicken, where the preservative effects of compound spice extracts, including tea tree oil, were evaluated. Kiarsi et al. [55] also noted that beef slices coated with 2% NEO and sage seed mucilage did not reach the maximum total plate count (TPC) value for fresh meat throughout the 6-day storage period, while the control samples exceeded the limit on day 2. Similarly, Wang et al. [78] reported a significant reduction in the TPC of chicken meat treated with plant essential oil components from the thymol and carvacrol groups, compared to the control samples during storage.

#### 3.6.2. *Staphylococcus aureus* Count

The *S. aureus* count for the control samples ranged from 1.41 ± 0.01 to 2.81 ± 0.03 log CFU/g during 1–9 days of aerobic refrigerated storage, whereas the mean values for T1 and T2 ranged from 1.36 ± 0.01 to 1.17 ± 0.02 log CFU/g and 1.37 ± 0.01 to 1.22 ± 0.01 log CFU/g, respectively (Table 5). Although all test samples displayed a significant (*p* < 0.01) increase in the *S. aureus* count, both NEO- and TTEO-treated samples (T1 and T2) had a delayed growth and reduced *S. aureus* count. Such lower counts of *S. aureus* could be attributed to the antibacterial activity of NEO [51] and TTEO [77] essential oils against Gram-positive bacteria such as *S. aureus*, as previously described. In a similar study, Kiarsi et al. [55] observed that beef slices coated with different concentrations of 0.5%, 1%, 1.5%, or 2% NEO, along with sage seed mucilage, exhibited a reduced *S. aureus* count compared to control beef samples. Likewise, Ibrahim et al. [79] found that essential oils (thyme, clove, and garlic) were significantly effective in reducing the *S. aureus* count in artificially inoculated minced beef samples.

#### 3.6.3. *Escherichia coli* Count

The *E. coli* count for the control samples ranged from 1.94 ± 0.02 to 2.71 ± 0.02 log CFU/g, whereas the values for T1 and T2 ranged from 1.71 ± 0.02 to 1.84 ± 0.02 and 1.38 ± 0.01 to 1.31 ± 0.01 log CFU/g, respectively, during 1–9 days of aerobic refrigerated storage (Table 5). Both NEO and TTEO treatments significantly (*p* < 0.05) reduced the *E. coli* count over the 9-day storage period in control, whereas EO treatments caused a growth delay. The lower count and growth delay observed in treatments T1 and T2 can be attributed to the antibacterial activity of NEO against *E. coli*, as established in other studies [51,80], and the antibacterial activity of TTEO against *E. coli* and other pathogenic bacteria [77].

These findings align with the reports of Kiarsi et al. [55], which demonstrated that coating beef slices with different concentrations of NEO, along with sage seed mucilage, reduced the *E. coli* count, compared to control samples. Similarly, Karam et al. [81] reported that a thymol and carvacrol mixture (1:1) at 0.4% and 0.8% in marinating solution reduced the *E. coli* count in fresh chicken breast fillets by up to 0.8 log CFU/g during refrigerated storage at 4 °C for 21 days. Comparable observations were also recorded in fresh pork meat with ginger EO [82] and a mixture of Mexican oregano and basil EOs [83].

#### 3.6.4. Total Coliform Count

Coliforms, a group of microorganisms, are recognized as indicators of hygienic quality in meat and meat products [2]. In this study, the total coliform count for the control samples ranged from 2.84 ± 0.18 to 3.91 ± 0.07 log CFU/g, whereas the values for T1 and T2 were from 1.65 ± 0.03 to 2.17 ± 0.04 log CFU/g and 1.55 ± 0.03 to 1.12 ± 0.03 log CFU/g, respectively, during 1–9 days of aerobic refrigerated storage (Table 5). Over the 9-day storage period, all test samples displayed a significant (*p* < 0.05) increase in the count; however, EO-treated samples (T1-NEO and T2-TTEO) had delayed growth compared to control. This phenomenon could be attributed to the bacteriostatic and bactericidal effects of the essential oils [80]. A significant reduction in the coliform count was reported in poultry meat with the use of garlic and thyme essential oils [84]. In another study, the rate of increase in coliform counts of chicken fillets was found to be significantly (*p* < 0.05) lower in coated samples with chitosan and *Artemisia fragrans* EO at 1500 ppm [54].

#### 3.6.5. *Salmonella* Count

During the stages of slaughter, processing, and marketing, raw fresh meat is susceptible to *Salmonella* contamination, which can lead to bacterial infections and food poisoning. Thus, the effective monitoring of *Salmonella* is paramount for preventing and managing foodborne illnesses, in order to safeguard human health and well-being [78]. During the aerobic refrigerated storage of 9 days, *Salmonella* spp. was detected neither in control nor treated chicken fillets with NEO and TTEO (Table 5). The absence of *Salmonella* in the meat samples could be attributed to good handling procedures during meat sample preparation and hygienic laboratory conditions [85].

In partial agreement with the present study, Cui et al. [86] observed that chicken cubes inoculated with *Salmonella* strains showed a reduction in the population of *S. enteritidis* in chicken meat by 99.53%, after treatment using tea tree oil liposomes/chitosan nanofibers, compared to the control samples. Similarly, Wang et al. [78] reported that plant essential oil components, thymol and carvacrol, reduced the *S. enteritidis* count in previously inoculated chicken meat during the first two days of storage. The count remained lower than the control samples until the end of the 6-day storage period.

### 3.7. Sensory Qualities of Chicken Fillets

The findings of the current study indicate that the inclusion of NEO and TTEO in raw chicken fillets effectively enhanced sensory attributes, compared to the control samples during refrigerated storage. There was a significant (*p* < 0.05) decrease in sensory scores for attributes such as color and odor for all test samples as the storage days progressed (Table 6). Regarding the color attribute, there were no significant differences (*p* > 0.05) between control, T1, and T2 until day 3, whereas the values were consistently higher for T1 and T2 during the 5th, 7th, and 9th days of storage study. Concerning the odor attribute, a significant difference (*p* < 0.05) was observed between the control samples and treatments T1 and T2 from day 3 of storage. Mean odor scores were significantly higher for the treatment samples, with slightly higher scores noted for samples treated with TTEO. Furthermore, the mean color and odor scores for the control samples significantly (*p* < 0.05) declined from day 5 of the storage period. The increased microbial counts observed in the control samples without essential oils could potentially explain their premature spoilage. Additionally, the generation of lipid oxidation by-products and the release of ammonia due to microbial protein breakdown might have contributed to the development of unpleasant odors, potentially accounting for the low rating of untreated samples by the 5th day of storage.

The color scores for control, T1, and T2 ranged from 4.38 ± 0.02 to 1.75 ± 0.22, 4.42 ± 0.13 to 2.43 ± 0.03, and 4.33 ± 0.17 to 2.43 ± 0.09, respectively, from day 1 to day 9 of refrigerated storage (Table 6). Similarly, the odor scores for control, T1, and T2 ranged from 4.41 ± 0.09 to 1.02 ± 0.02, 4.55 ± 0.07 to 2.07 ± 0.04, and 4.62 ± 0.02 to 2.19 ± 0.02, respectively, from day 1 to day 9 of refrigerated storage (Table 6). In a related study, Šojić et al. [87] found that cooked sausages treated with 20 ppm NEO had a significantly better aroma (*p*< 0.05) compared to the control samples after 60 days of storage. Similar observations were reported by Cui et al. [86], where chicken cubes treated with tea tree oil liposomes/chitosan nanofibers maintained their color, flavor, juiciness, and overall appeal, almost unchanged, when stored for 4 days at both 4 °C and 12 °C. Furthermore, Wang et al. [78] treated chicken meat with plant essential oil components such as thymol, carvacrol, and cinnamaldehyde, and noted significant differences in color, odor, viscosity, and overall acceptability (*p* < 0.05) on the 4th day of storage between the treated groups and the control groups, with higher scores observed for the treated groups. Additionally, Zdunczyk et al. [88] reported that the application of oregano essential oil to the surface of pork did not affect meat taste intensity, although the aroma and taste of oregano EO were noticeable to consumers. Talebi et al. [89] evaluated the effect of active polylactic acid in ground beef for 12 days at 4 ± 1 °C, observing that the presence of a lower concentration of *Bunium percicum* dried leaves EO resulted in significantly better outcomes compared to the control group regarding the odor parameter.

## 4. Conclusions

This study identified the chemical compositions of NEO and TTEO using gas chromatography–mass spectrometry (GC-MS), and evaluated their antioxidant and antimicrobial properties in extending the shelf life of chicken fillets stored at 4 ± 1 °C for a period of 9 days. Shelf-life investigations involved analyzing the physico-chemical, microbiological, and sensory qualities of the raw chicken fillets, until visible changes in their characteristics began to emerge. The deterioration of meat samples in the control group was observed within 5 days, coinciding with an increase in the total viable count, lipid oxidation, and a reduction in sensory attributes. However, raw chicken fillets dipped in or coated with 1% nutmeg or tea tree essential oils demonstrated the ability to be stored for 7 days at 4 ± 1 °C. From this study, it is concluded that essential oils derived from nutmeg and tea tree oil can be used as natural preservatives in extending the shelf life and ensuring the safety and quality of raw chicken fillets.

## Figures and Tables

**Table 1:** Total phenolics, total flavonoid content, DPPH, and ABTS radical scavenging activity of nutmeg and tea tree essential oils (mean ± standard error).

Parameters	Nutmeg Essential Oil	Tea Tree Essential Oil
Total phenolics content (mg GAE/g)	64.51 ± 0.82	78.21 ± 0.75
Total flavonoid content (mg QE/g)	9.38 ± 0.27	17.29 ± 0.31
DPPH (IC50 µg/mL)	2.94 ± 0.18	12.68 ± 0.24
ABTS (IC50 µg/mL)	1.26 ± 0.22	8.62 ± 0.55

**Table 2:** Effect of essential oils on proximate composition of chicken fillets.

Treatments	Moisture%	Protein%	Fat%	Ash%
Control	73.77 ± 0.73	21.55 ± 0.43	2.52 ± 0.14	1.45 ± 0.28
T1-NEO	73.98 ± 0.71	21.61 ± 0.67	2.49 ± 0.04	1.41 ± 0.26
T2-TTEO	73.92 ± 0.51	21.63 ± 0.36	2.54 ± 0.02	1.49 ± 0.35

Control—no essential oil; T1-NEO—1% nutmeg essential oil; T2-TTEO—1% tea tree essential oil, n = 6.

**Table 3:** Effect of essential oils on physico-chemical parameters of raw chicken fillets.

Treatments	Refrigeration Storage (Days)				
	1	3	5	7	9
			pH		
Control	5.72 ± 0.01 ^eA^	5.80 ± 0.03 ^dA^	5.92 ± 0.02 ^cA^	6.17 ± 0.02 ^bA^	7.42 ± 0.04 ^aA^
T1-NEO	5.73 ± 0.02 ^dA^	5.75 ± 0.02 ^cdA^	5.80 ± 0.02 ^bcB^	5.83 ± 0.01 ^abB^	6.87 ± 0.01 ^aB^
T2-TTEO	5.71 ± 0.02 ^dA^	5.74 ± 0.02 ^cdA^	5.81 ± 0.01 ^bcB^	5.83 ± 0.01 ^abB^	6.86 ± 0.01 ^aB^
			WHC (%)		
Control	73.84 ± 2.06	70.72 ± 2.07	65.79 ± 1.52	63.50 ± 0.92	61.35 ± 0.55
T1-NEO	73.75 ± 1.12	71.59 ± 1.83	69.74 ± 1.47	68.28 ± 0.98	67.04 ± 1.40
T2-TTEO	73.80 ± 0.48	72.41 ± 1.78	71.09 ± 1.21	68.96 ± 1.06	68.21 ± 1.60

Control—no essential oil; T1-NEO—1% nutmeg essential oil; T2-TTEO—1% tea tree essential oil, n = 6. Mean ± SE with different superscript row-wise (a–e) and column-wise (a–c) differ significantly (*p* < 0.05).

**Table 4:** Effect of essential oils on color profile of chicken fillets.

Treatments	Refrigeration Storage (Days)				
	1	3	5	7	9
Lightness (L*)					
Control	56.98 ± 2.06 ^a^	54.23 ± 1.76 ^a^	49.72 ± 0.66 ^bB^	46.39 ± 0.38 ^bcB^	42.70 ± 0.44 ^cB^
T1-NEO	56.78 ± 1.95	55.24 ± 1.52	54.74 ± 1.18 ^A^	53.50 ± 1.83 ^A^	52.53 ± 1.70 ^A^
T2-TTEO	55.94 ± 2.02	55.23 ± 2.42	54.66 ± 1.38 ^A^	53.79 ± 0.62 ^A^	52.30 ± 1.43 ^A^
Redness (a*)					
Control	8.65 ± 0.53 ^a^	8.36 ± 0.25 ^a^	6.99 ± 0.22 ^bB^	4.86 ± 0.34 ^bB^	2.91 ± 0.25 ^bB^
T1-NEO	8.59 ± 0.34 ^a^	8.38 ± 0.35 ^a^	8.18 ± 0.35 ^aA^	7.80 ± 0.26 ^aA^	6.38 ± 0.28 ^bA^
T2-TTEO	8.42 ± 0.32 ^a^	8.22 ± 0.34 ^a^	8.10 ± 0.37 ^aA^	8.01 ± 0.28 ^aA^	7.09 ± 0.21 ^bA^
Yellowness (b*)					
Control	9.67 ± 0.21	10.17 ± 0.31	10.92 ± 0.25	12.01 ± 0.99	12.76 ± 0.61
T1-NEO	9.65 ± 0.30	9.88 ± 0.40	10.16 ± 0.44	10.53 ± 0.22	10.74 ± 0.57
T2-TTEO	9.58 ± 0.26	9.79 ± 0.28	9.92 ± 0.28	10.20 ± 0.36	10.72 ± 0.32

Control—no essential oil; T1-NEO—1% nutmeg essential oil; T2-TTEO—1% tea tree essential oil, n = 6. Means with different superscript row-wise (a–e) and column-wise (a–c) differ significantly (*p* < 0.05).

**Table 5:** Effect of essential oils on microbiological counts of chicken fillets.

Treatments	Refrigeration Storage (Days)				
	1	3	5	7	9
Total viable count (log CFU/g)					
Control	4.91 ± 0.36 ^cA^	5.68 ± 0.25 ^bA^	6.88 ± 0.12 ^aA^	7.94 ± 0.17 ^aA^	SS
T1-NEO	3.27 ± 0.23 ^bB^	3.68 ± 0.27 ^abB^	3.92 ± 0.4 ^abB^	4.26 ± 0.18 ^aB^	5.51 ± 0.18 ^a^
T2-TTEO	3.17 ± 0.26 ^bB^	3.37 ± 0.24 ^bB^	3.71 ± 0.2 ^bB^	3.91 ± 0.17 ^bB^	5.81 ± 0.37 ^a^
*Staphylococcus aureus* count (log CFU/g)					
Control	1.41 ± 0.01 ^eA^	1.65 ± 0.03 ^dA^	1.90 ± 0.01 ^cA^	2.27 ± 0.06 ^bA^	2.81 ± 0.03 ^aA^
T1-NEO	1.36 ± 0.01 ^cB^	1.33 ± 0.01 ^cB^	1.26 ± 0.01 ^bB^	1.21 ± 0.02 ^abB^	1.17 ± 0.02 ^aB^
T2-TTEO	1.37 ± 0.01 ^cAB^	1.35 ± 0.02 ^cB^	1.31 ± 0.01 ^bcC^	1.26 ± 0.01 ^abB^	1.22 ± 0.01 ^aB^
*Escherichia coli* count (log CFU/g)					
Control	1.94 ± 0.02 ^cA^	1.98 ± 0.14 ^cA^	2.32 ± 0.03 ^bA^	2.54 ± 0.03 ^aA^	2.71 ± 0.02 ^aA^
T1-NEO	1.71 ± 0.02 ^cB^	1.73 ± 0.02 ^bcA^	1.75 ± 0.03 ^abcB^	1.82 ± 0.04 ^abB^	1.84 ± 0.02 ^aB^
T2-TTEO	1.38 ± 0.01 ^C^	1.36 ± 0.02 ^B^	1.35 ± 0.03 ^C^	1.33 ± 0.03 ^C^	1.31 ± 0.01 ^C^
Total coliform count (log CFU/g)					
Control	2.84 ± 0.18 ^cA^	3.14 ± 0.10 ^bcA^	3.36 ± 0.03 ^bA^	3.57 ± 0.26 ^abA^	3.91 ± 0.07 ^aA^
T1-NEO	1.65 ± 0.03 ^bB^	1.68 ± 0.10 ^bB^	1.73 ± 0.02 ^bB^	1.77 ± 0.02 ^bB^	2.17 ± 0.04 ^aB^
T2-TTEO	1.55 ± 0.03 ^cB^	1.26 ± 0.02 ^bC^	1.17 ± 0.02 ^aC^	1.15 ± 0.01 ^aC^	1.12 ± 0.03 ^aC^
*Salmonella* spp. count (log CFU/g)					
Control	ND	ND	ND	ND	ND
T1-NEO	ND	ND	ND	ND	ND
T2-TTEO	ND	ND	ND	ND	ND

Control—no essential oil; T1-NEO—1% nutmeg essential oil; T2-TTEO—1% tea tree essential oil, n = 6; SS—sample spoiled; ND—not detected. Means with different superscript row-wise (a–e) and column-wise (a–c) differ significantly (*p* < 0.05).

**Table 6:** Effect of essential oils on sensory attributes of chicken fillets.

Treatments	Refrigeration Storage (Days)				
	1	3	5	7	9
Color					
Control	4.38 ± 0.02 ^a^	4.12 ± 0.16 ^ab^	3.92 ± 0.10 ^bB^	2.17 ± 0.07 ^cB^	1.75 ± 0.22 ^dB^
T1-NEO	4.42 ± 0.13 ^a^	4.43 ± 0.03 ^a^	4.34 ± 0.01 ^aA^	3.48 ± 0.20 ^bA^	2.43 ± 0.03 ^bA^
T2-TTEO	4.33 ± 0.17 ^a^	4.24 ± 0.13 ^a^	4.21 ± 0.12 ^aA^	3.57 ± 0.09 ^bA^	2.43 ± 0.09 ^bA^
Odor					
Control	4.41 ± 0.09 ^a^	3.81 ± 0.19 ^bB^	2.57 ± 0.06 ^cB^	1.91 ± 0.02 ^dB^	1.02 ± 0.02 ^eC^
T1-NEO	4.55 ± 0.07 ^a^	4.27 ± 0.02 ^bA^	4.18 ± 0.02 ^bA^	3.52 ± 0.05 ^cA^	2.07 ± 0.04 ^dB^
T2-TTEO	4.62 ± 0.02 ^a^	4.48 ± 0.03 ^bB^	4.27 ± 0.03 ^cA^	3.52 ± 0.04 ^dA^	2.19 ± 0.02 ^eA^

Control—no essential oil; T1-NEO—1% nutmeg essential oil; T2-TTEO—1% tea tree essential oil, n = 6. Means with different superscript row-wise (a–e) and column-wise (a–c) differ significantly (*p* < 0.05).

## Data Availability

All data are presented in the article/Supplementary Materials, and further inquiries can be directed to the corresponding authors.

## Supplementary Materials

The following supporting information can be downloaded at: https://www.mdpi.com/article/10.3390/foods13244139/s1, Table S1: Chemical composition of Nutmeg essential oil; Table S2: Chemical composition of Tea tree essential oil.
