# Supplementary material for: Effect of Nutmeg (Myristica fragrans) and Tea Tree (Melaleuca alternifolia) Essential Oils on the Oxidative and Microbial Stability of Chicken Fillets During Refrigerated Storage
Source: Foods. 2024 Dec 20;13(24):4139. doi: 10.3390/foods13244139 (PMC11675315; doi:10.3390/foods13244139)
Supplement: Supplementary file 1 [file foods-13-04139-s001.zip › foods-3292734-supplementary.pdf]

Supplementary Materials

Table S1. Chemical composition of Nutmeg essential oil.

Peak#	Retention Time	Area%	Name
1	3.175	2.09	.alpha.-Phellandrene
2	3.246	0.62	3-Carene
3	3.294	0.28	(+)-4-Carene
4	3.378	11.54	o-Cymene
5	3.439	18.44	Cyclobutane, 1,2-bis(1-methylethenyl)-, trans-
6	3.780	9.34	.gamma.-Terpinene
7	3.908	0.72	Bicyclo[3.1.0]hexan-2-ol, 2-methyl-5-(1-methylethyl)-, (1.alpha.,2.beta.,5.alpha.)-
8	4.205	0.43	Cyclohexene, 1-methyl-4-(1-methylethylidene)-
9	4.268	0.37	
10	4.365	0.84	Bicyclo[3.1.0]hexan-2-ol, 2-methyl-5-(1-methylethyl)-, (1.alpha.,2.beta.,5.alpha.)-
11	4.549	0.18	Bicyclo[3.1.1]hept-3-en-2-ol, 4,6,6-trimethyl-, [1S-(1.alpha.,2.beta.,5.alpha.)]-
12	5.125	0.33	Bicyclo[3.1.1]heptan-3-ol, 6,6-dimethyl-2-methylene-, [1S-(1.alpha.,3.alpha.,5.alpha.)]-
13	5.821	1.84	3-Cyclohexen-1-ol, 4-methyl-1-(1-methylethyl)-, (R)-
14	6.071	2.16	.alpha.-Terpineol
15	6.214	0.83	(-)-Myrtenol
16	8.481	0.74	Safrole
17	10.545	1.07	2,6-Octadien-1-ol, 3,7-dimethyl-, acetate, (Z)-
18	11.146	0.53	.alpha.-ylangene
19	11.726	0.66	Vanillin
20	11.849	1.51	Benzene, 1,2-dimethoxy-4-(1-propenyl)-
21	12.039	0.46	trans-Isoeugenol
22	12.583	2.86	Caryophyllene
23	13.420	6.73	trans-Isoeugenol
24	14.993	0.56	Benzene, 1,2-dimethoxy-4-(1-propenyl)-
25	15.890	4.06	1,3-Benzodioxole, 4-methoxy-6-(2-propenyl)-
26	17.075	10.32	1,2-Dimethoxy-4-(2-methoxy-1-propenyl)benzene
27	18.124	0.61	Caryophyllene oxide
28	20.376	1.19	Isoelemicin
29	24.383	0.95	Tetradecanoic acid
30	30.468	1.94	(E,E,E)-3,7,11,15-Tetramethylhexadeca-1,3,6,10,14-pentaene
31	31.546	0.66	(E,E,E)-3,7,11,15-Tetramethylhexadeca-1,3,6,10,14-pentaene
32	43.421	1.66	.alpha.-Terpinyl isovalerate
33	44.655	3.27	.alpha.-Terpinyl isovalerate
34	49.949	1.29	Bicyclo[2.2.1]heptan-2-ol, 1,5,5-trimethyl-
35	50.217	1.06	Bicyclo[2.2.1]heptan-2-ol, 1,5,5-trimethyl-
36	51.211	1.62	Thiazolo[3,2-a]benzimidazol-3(2H)-one, 2-(2-fluorobenzylideno)-7,8-dimethyl-

37	51.338	2.51	Phenol, 4-[2,3-dihydro-7-methoxy-3-methyl-5-(1-propenyl)-2-benzofuranyl]-2-methoxy-
38	54.521	2.51	Isoquinoline, 1,2,3,4-tetrahydro-8-amino-2-methyl-4-phenyl-
39	54.768	0.92	2-Hydroxy-4-isopropyl-7-methoxytropone
40	56.092	0.81	1-Phosphacyclopent-2-ene, 1,2,3-triphenyl-5-dimethylmethylene
41	57.199	1.06	2-Hydroxy-4-isopropyl-7-methoxytropone
	**Total**	**100.00**	

Table S2. Chemical composition of Tea tree essential oil.

**Peak#**	**Retention Time**	**Area%**	**Name**
1	3.289	5.50	(+)-4-Carene
2	3.369	6.18	Benzene, 1-methyl-3-(1-methylethyl)-
3	3.430	14.82	Cyclobutane, 1,2-bis(1-methylethenyl)-, trans-
4	3.486	9.55	Eucalyptol
5	3.771	11.26	γ-Terpinene
6	4.197	2.59	Cyclohexene, 1-methyl-4-(1-methylethylidene)-
7	5.869	44.42	3-Cyclohexen-1-ol, 4-methyl-1-(1-methylethyl)-, (R)-
8	6.074	4.99	L-.alpha.-Terpineol
9	6.206	0.60	Cyclohexanol, 1-methyl-4-(1-methylethylidene)-
10	8.050	0.09	Ethanone, 1-[2-methyl-5-(1-methylethenyl)cyclopentyl]-, (1.alpha.,2.alpha.,5.beta.)-
	**Total**	**100.00**	
